# Learning from Embryogenesis—A Comparative Expression Analysis in Melanoblast Differentiation and Tumorigenesis Reveals miRNAs Driving Melanoma Development

**DOI:** 10.3390/jcm10112259

**Published:** 2021-05-24

**Authors:** Lisa Linck-Paulus, Lisa Lämmerhirt, Daniel Völler, Katharina Meyer, Julia C. Engelmann, Rainer Spang, Norbert Eichner, Gunter Meister, Silke Kuphal, Anja Katrin Bosserhoff

**Affiliations:** 1 Institute of Biochemistry, Friedrich-Alexander-University Erlangen-Nürnberg, 91054 Erlangen, Germany; lisa.linck@fau.de (L.L.-P.); lisa.laemmerhirt@fau.de (L.L.); daniel.voeller@biontech.de (D.V.); silke.kuphal@fau.de (S.K.); 2 Institute of Functional Genomics, University of Regensburg, 93053 Regensburg, Germany; katharina-meyer@gmx.de (K.M.); rainer.spang@klinik.uni-regensburg.de (R.S.); 3 Department of Marine Microbiology and Biogeochemistry, NIOZ Royal Netherlands Institute for Sea Research, 1790 AB Den Burg, The Netherlands; julia.engelmann@nioz.nl; 4 Department of Biochemistry I, University of Regensburg, 93053 Regensburg, Germany; norbert.eichner@vkl.uni-regensburg.de (N.E.); gunter.meister@vkl.uni-regensburg.de (G.M.)

**Keywords:** miRNAs, melanoma, embryogenesis, melanoblasts

## Abstract

Malignant melanoma is one of the most dangerous tumor types due to its high metastasis rates and a steadily increasing incidence. During tumorigenesis, the molecular processes of embryonic development, exemplified by epithelial–mesenchymal transition (EMT), are often reactivated. For melanoma development, the exact molecular differences between melanoblasts, melanocytes, and melanoma cells are not completely understood. In this study, we aimed to identify microRNAs (miRNAs) that promote melanoma tumorigenesis and progression, based on an in vitro model of normal human epidermal melanocyte (NHEM) de-differentiation into melanoblast-like cells (MBrCs). Using miRNA-sequencing and differential expression analysis, we demonstrated in this study that a majority of miRNAs have an almost equal expression level in NHEMs and MBrCs but are significantly differentially regulated in primary tumor- and metastasis-derived melanoma cell lines. Further, a target gene analysis of strongly regulated but functionally unknown miRNAs yielded the implication of those miRNAs in many important cellular pathways driving malignancy. We hypothesize that many of the miRNAs discovered in our study are key drivers of melanoma development as they account for the tumorigenic potential that differentiates melanoma cells from proliferating or migrating embryonic cells.

## 1. Introduction

Malignant melanoma is an aggressively metastatic tumor with a high incidence that has been increasing for years [1]. Especially patients with an advanced metastatic tumor stage still have a poor outcome [2]. Interestingly, melanoma has molecular features that are not found in other tumors [3]. Two aspects are of particular importance during melanoma development:

1. Melanoblasts, the precursors of melanocytes derived from the neural crest, exhibit several mechanisms during embryogenesis usually known from tumor cells, e.g., they actively migrate, adapt to different cellular environments, and “invade” the epidermis [4,5].
2. Melanoma cells can build stem-cell-like subpopulations that have the ability to differentiate into several cell lineages such as neural, mesenchymal, and endothelial cells [6,7,8].

These characteristic features show that melanoma cells can re-activate the pathways of neural crest differentiation and melanoblast migration, and thus reflect the high plasticity of malignant melanoma cells.

The neural crest, a transient component of the ectoderm, is located between the neural tube and epidermis during embryonal neural tube formation. Neural crest cells migrate during or shortly after neurulation, an embryological event characterized by the closure of the neural tube. Because of their great importance, they have been called the fourth germinal layer. Neural crest cells can differentiate into various cell types such as neurons and glial cells of the autonomic nervous system, some skeletal elements, and melanocytes [9]. There are two main migration pathways of neural crest cells: the ventral pathway and the dorsolateral pathway. Melanoblasts mainly migrate through the dorsolateral pathways between the somites and the ectoderm to their target region, the epidermis, where they then differentiate to melanocytes [4,5,10].

In addition to the high differentiation plasticity, malignant melanoma is characterized by being the tumor type with the highest mutation rate [11]. To date, however, only a few “drivers” of tumor development have been described in comparison to other cancers and all of those frequently mutated driver genes seem only to play a secondary role in the initiation of tumor metastasis [12].

microRNAs (miRNAs) are small non-coding RNAs that can regulate gene expression on a post-transcriptional level [13]. They are transcribed in the nucleus as long, double-stranded precursor molecules containing a characteristic stem-loop structure. Two mature, single-stranded miRNAs, which are complementary to each other and about 21 nucleotides long, are processed from the precursor by an intracellular enzymatic cascade. The location in the precursor molecule discriminates the annotation of the mature miRNA: the miRNA in the precursor arm with the 5′ end is called 5p, and the miRNA in the 3′ arm is called 3p [14]. One mature miRNA molecule binds to one of the four human Argonaute proteins to form the RNA-induced silencing complex (RISC). The RISC identifies target messenger RNAs (mRNAs) via complementary base pairing, interferes with their translation, and simultaneously mediates decay of the target mRNA by hydrolytic cleavage or via cellular degradation mechanisms [15]. miRNAs are deeply involved in the regulation of all important cellular processes and are the main contributor to the formation and progression of cancer [16]. In melanoma, many studies show that the expression of miRNAs is deregulated compared to normal human epidermal melanocytes and that deregulated miRNA expression is linked to important processes affecting tumor formation and progression [17,18,19,20]. The role of miRNAs during melanoblast differentiation and their involvement in de-differentiation processes that may drive tumor development, such as migration and invasion, is not well understood.

The aim of this study was to identify miRNAs that drive melanoma development and progression via a comparative analysis of miRNA expression in melanoblasts, differentiated melanocytes, and melanoma cells from primary tumors and metastases.

## 2. Materials and Methods

### 2.1. Cultivation of Melanocytes and De-Differentiation into Melanoblast-Related Cells

Normal human epidermal melanocytes (NHEM) were obtained from PromoCell (Heidelberg, Germany) or Lonza (Basel, Switzerland) and were derived from human neonatal foreskin tissue of Caucasian donors. The melanocytes were grown either in melanocyte serum-free M2 medium without PMA (phorbol myristate acetate) from PromoCell (Heidelberg, Germany) or in melanocyte serum-free medium with PMA from Lonza (Basel, Switzerland) at 37 °C and 5% CO_2_. For de-differentiation, NHEM were grown for three passages and subsequently cultivated in a special melanoblast growth medium for 7 or 14 days to de-differentiate into melanoblast-related cells (MBrCs): MCBD 153 medium (Sigma-Aldrich, Steinheim, Germany) containing 8% chelated fetal bovine serum (FBS), 2% normal FBS (PAA Laboratories, Pasching, Austria), 2 mM glutamine, 1.66 ng/mL cholera toxin B, 10 ng/mL SCF (Sigma-Aldrich, Steinheim, Germany), 100 nM endothelin-3 and 2.5 ng/mL bFGF. Chelated FBS was prepared by mixing 1.2 g of Chelex-100 (Sigma-Aldrich, Steinheim, Germany) per 40 mL of FBS for 1.5 h at 4 °C with gentle stirring. One sub-group of cells continued growing in PromoCell M2 medium (labeled as melanocytes in this study). The de-differentiation procedure of melanocytes to MBrCs is published by Cook et al. [21].

### 2.2. Melanoma Cell Culture

The melanoma cell lines Mel Juso (RRID:CVCL_1403), Mel Ei (RRID:CVCL_3978), Mel Wei (RRID:CVCL_3981), and Mel Ho (RRID:CVCL_1402) [22] were derived from primary cutaneous melanomas, whereas Mel Ju (RRID:CVCL_3979), Mel Im (RRID:CVCL_3980) [22], Hmb2 (RRID:CVCL_6646) [23], A375 (RRID:CVCL_0132) [24], 1205Lu (RRID:CVCL_5239) [25], and 501 Mel (RRID:CVCL_4633) [26] were derived from metastases of malignant melanomas. The cells were maintained in DMEM or RPMI-1640 medium (Sigma-Aldrich, Steinheim, Germany) supplemented with penicillin (400 units/mL), streptomycin (50 mg/mL), and 10% FBS (Sigma-Aldrich, Steinheim, Germany). Only RPMI was additionally supplemented with 0.2% sodium bicarbonate (Sigma-Aldrich, Steinheim, Germany). The melanoma cells were incubated in a humidified atmosphere containing 8% CO_2_ at 37 °C in T75 cell culture flasks (Corning Incorporated, New York, NY, USA). They were split at a ratio of 1:5 every 3 days.

### 2.3. microRNA-Sequencing and Bioinformatic Sequence Data Analysis

microRNA-sequencing was performed on four different samples of NHEM from different passages, two independent replicates of MBrCs and the melanoma cell lines Mel Wei, Mel Ei, Mel Juso, Mel Ju, Mel Im, and Hmb2. Performance of sample preparation, miRNA profiling, and sequencing are described elsewhere [17]. miRNA-sequencing data of melanoma cells and NHEMs are already published [17]. For this study, all miRNA sequencing data were re-analyzed using current software and databases: preprocessing and counting against human miRNA listed in the miRbase database (http://www.mirbase.org; version 22 March 2018) was performed using the “miRDeep2” package [27] running with standard parameters as part of the Galaxy environment [28]. After transferring the resulting raw miRNA counts to R, normalization and differential expression analysis were performed using the “Deseq2” package [29].

The heatmaps of differentially expressed miRNAs were designed with Microsoft Excel^®^ using a 3-color scale with red, white, and blue representing the minimum, median (50th percentile), and maximum, respectively. The colors encode log2FoldChange from Deseq2, and the highest and the lowest log2fold change are indicated respectively.

Principal component analysis (PCA) was performed using https://maayanlab.cloud/biojupies (accessed on 13 January 2021) [30]. Venn diagrams were calculated using the “VennDiagram” package in R and were illustrated with the venn.diagram function. The overlap was calculated using the calculate.overlap function. Figures were created using CorelDRAW^®^ 2017 (64-Bit, Corel Corporation, Ottawa, ON, Canada).

Analysis of predicted target genes of miRNAs was performed using http://www.targetscan.org/vert_72, release 7.2 March 2018 [31]. Gene ontology enrichment analysis of predicted targets was performed with http://geneontology.org, release 1 January 2021 [32]. The STRING network analysis of miRNA target genes was performed using https://string-db.org, version 11.0b October 2020 [33] and presented using the stringApp in Cytoscape version 3.8.2 [34]. The STRING network shows known protein–protein associations from curated databases or experimentally determined, predicted interactions from gene neighborhood, gene fusions or co-occurrence and text mining, and co-expression or homology-based interactions. The minimum required interaction score was 0.4.

### 2.4. Gene Expression Microarray and Gene Set Enrichment Analysis (GSEA) of miRNA Target Genes

Gene expression microarray analysis was performed with three different replicates of MBrCs and the respective NHEM in the melanoma cell lines Mel Ho, A375, 501 Mel, and 1205 Lu. RNA was isolated with Rneasy^®^ Mini Kit of Qiagen (Hilden, Germany) and was reverse-transcribed followed by hybridization to the Human Gene 1.0 ST Expression Array from Affymetrix (Santa Clara, CA, USA). Sample processing and Affymetrix microarray hybridization were carried out at the Genomics Core Unit: Center of Excellence for Fluorescent Bioanalytics (KFB, University of Regensburg, Regensburg, Germany). Signal intensities were summarized to Affymetrix probe_set level using ‘rma’ within the Bioconductor package oligo [35], and packages pd.hugene.1.0.st.v1 and hugene10sttranscriptcluster [36]. Probe_sets with an Entrez identifier were retained (21,995 genes) for further analyses. We used the non-parametric empirical Bayes approach of Combat [37] to remove the donor effects of MBrCs and the corresponding NHEM and ranked differentially expressed genes between melanoblasts and melanocytes versus melanoma cell lines by fold change using limma [38]. This ranked gene list was used for Gene Set Enrichment Analysis with the GSEA software version 4.0.3 (https://www.gsea-msigdb.org/gsea/index.jsp) [39,40]. For GSEA analysis, the C3 collection of the molecular signature database was used (MSigDB version 7.2, https://www.gsea-msigdb.org/gsea/msigdb/collection_details.jsp#MIRDB) with special attention to the miRDB gene set collection consisting of MirTarget high-confidence predicted human miRNA target genes [41] and the MIR Legacy geneset containing genes with 7-nucleotide miRNA-binding motifs of miRNAs cataloged in v7.1 of miRBase (http://www.mirbase.org, version 7.1, October 2005). For calculation of the false discovery rate (FDR), a pre-ranked GSEA analysis using one single gene set with targets of the respective miRNA was performed.

### 2.5. Isolation and Reverse Transcription of miRNAs from Mammalian Cells

The isolation and purification of miRNAs were performed using the miRNeasy Mini Kit from Qiagen (Hilden, Germany) according to the manufacturer. For the reverse transcription, the miScript II RT Kit from Qiagen (Hilden Germany) was used. In this study, 500 ng of miRNA were reversed transcribed into micDNA according to the manufacturer’s specification.

### 2.6. Quantitative RT-PCR with miRNA

The qRT-PCR was performed on a *LightCycler*^®^ 480 System from Roche (Mannheim, Germany). For the determination of the relative expression of miRNAs in melanocytes (at least *n* = 3), MBrCs (at least *n* = 3) and different melanoma cell lines (at least *n* = 9), from primary melanoma and melanoma metastasis, the *miScript SYBR*^®^ *Green* PCR Kit of Qiagen (Hilden, Germany) was used. The snRNA U6 was taken as a reference for the relative determination of miRNAs. For qRT-PCR, a 25 µL reaction was used containing 2 µL of transcribed miRNA, 12.5 µL of miScript SYBR Green, 2.5 µL of the respective miScript Primer Assay (Qiagen, Hilden, Germany), 2.5 µL of miScript Universal primer (Qiagen), and 5.5 µL RNase-free water. The following PCR program containing 60 cycles was used: 4.4 °C/s to 95 °C 15 min (initial denaturation); 2.2 °C/s to 94 °C 15 s (denaturation); 2.2 °C/s to 55 °C 30 s (annealing); 4.4 °C/s to 70 °C 30 s (amplification); 4.4 °C/s to 72 °C 1 s (measurement); 4.4 °C/s to 95 °C 5 s, 2.2 °C/s to 65 °C 1 min, and 0.11 °C/s to 97 °C (melting point analysis); and 2.2 °C/s to 40 °C 30 s (end). The PCR reaction was evaluated by melting curve analysis. Statistical analysis was performed via the software GraphPad Prism 5.04 (Version 5.0.4.533, GraphPad Software Inc., San Diego, CA, USA).

## 3. Results

### 3.1. miRNAs Differentially Expressed Only in Melanoma Cells but Not in NHEMs and Melanoblasts Drive Tumor Development

To identify the unknown miRNAs and their respective target molecules, which are important for the development or progression of melanoma, we followed the idea of “learning from embryology”. To deduce tumor-relevant molecular mechanisms from comparison with embryonic development, two hypotheses are plausible:

1. Genes and miRNAs, which are equally expressed in melanoma and melanoblasts but differently in melanocytes, are the relevant decisive factors for melanoma development; or
2. genes and miRNAs differentially expressed in melanoma compared to melanocytes and melanoblasts are key drivers of melanoma development and progression because these are stabilizing the tumor phenotype. This second hypothesis implicates that hypothesis 1 “only” focuses on genes, which are involved in differentiation/dedifferentiation processes but not in forcing tumor progression.

Using an experimental system first described by Rick Sturm’s group [21], we de-differentiated melanocytes into melanoblast-related cells (MBrC) using a cytokine cocktail (SCF, End-3, and bFGF) [5]. miRNA expression of MBrCs was compared to the respective normal human epidermal melanocytes (NHEMs) and melanoma cell lines derived from primary tumors (Mel Wei, Mel Ei, and Mel Juso) as well as from melanoma metastases (Mel Ju, Mel Im, and Hmb2) by miRNA sequencing.

**Figure 1:**
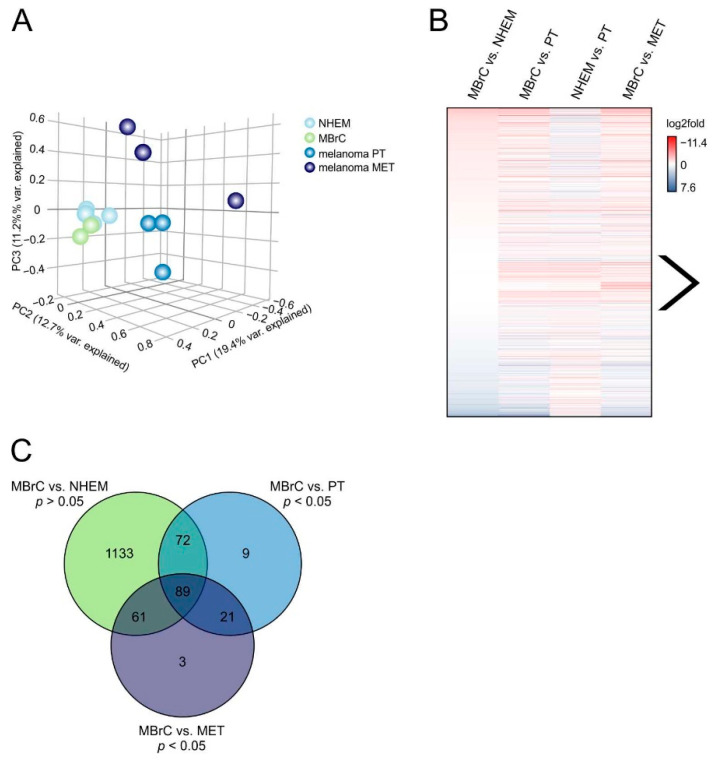
miRNA sequencing in NHEMs, MBrCs, and melanoma cell lines reveals a group of miRNAs, which are strongly regulated only in melanoma. (**A**) Principal component analysis of miRNA-seq data plot of the first three principal components (PCs). Samples with related gene expression profiles are closer in the three-dimensional embedding. Each sphere represents an RNA-seq sample, and replicates are shown in the same color: NHEM (*n* = 4), MBrC (*n* = 2), melanoma primary tumor (PT) cell lines Mel Juso, Mel Wei and Mel Ei, melanoma metastasis (MET) cell lines Mel Im, Mel Ju and Hmb2. (**B**) Differential gene expression analysis of miRNA-seq data in the indicated sample pairs is shown as a heatmap of log2fold values. The arrow indicates miRNAs, which are equally expressed in MBrCs and NHEMs but strongly upregulated in melanoma cell lines. (**C**) Venn diagram of miRNAs that are not significantly differentially expressed in MBrCs vs. NHEMs (*p*-value > 0.05) but significantly differentially expressed (*p*-value < 0.05) in MBrCs vs. melanoma primary tumor (PT) or metastasis-derived (MET) melanoma cell lines.

We first analyzed the data using a principal component analysis (PCA). The PCA shows similar miRNA expression for NHEMs and MBrCs but considerable differences in melanoma (Figure 1A). Interestingly, the PCA supports the second hypothesis. It indicates that miRNAs are mainly equally expressed during melanoblast to melanocyte development but differentially regulated during tumor progression and thus may drive malignancy.

To further analyze this hypothesis, differential expression analysis of miRNAs was performed between all groups (MBrCs vs. NHEMs, MBrCs vs. primary tumor cell lines (PT), NHEMs vs. primary tumor-derived cell lines, and MBrCs vs. metastasis-derived cell lines (MET)) and illustrated in a heatmap (Figure 1B). The figure shows a noteworthy group of miRNAs, which are equally expressed in MBrCs and NHEMs, but strongly upregulated in melanoma cell lines (Figure 1B, indicated by arrow). These specific miRNAs follow the second hypothesis for learning from embryology and their in-depth analyses could provide molecular information about melanoma development.

To analyze this group of miRNAs in more detail, we filtered miRNA expression data for only miRNAs that were significantly differentially expressed (*p*-value < 0.05) in MBrCs vs. melanoma (primary tumor or metastasis-derived melanoma cell lines) but simultaneously not significantly regulated in MBrCs vs. NHEMs (*p*-value > 0.05). The miRNAs that fulfill these conditions are visualized in a Venn diagram (Figure 1C). The analysis provided 89 miRNAs that were significantly regulated in both, primary tumor and metastasis-derived melanoma cell lines, compared to MBrCs and NHEMs.

**Figure 2:**
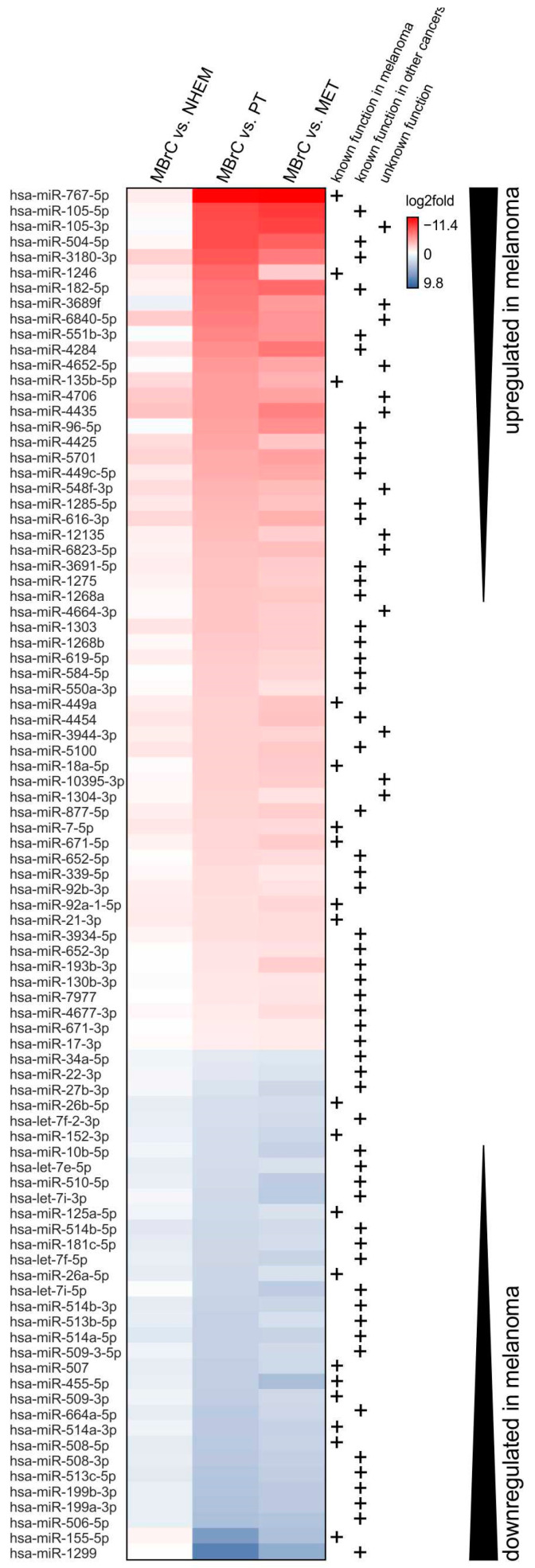
Significantly differentially expressed miRNAs in melanoma cells. Heatmap of log2fold values of all miRNAs that were not regulated in MBrCs vs. NHEMs (*p* > 0.05), but differentially expressed (*p* < 0.05) in MBrCs vs. melanoma primary tumor (PT), and MBrCs vs. melanoma metastasis-derived (MET) cell lines. miRNAs, which were upregulated in melanoma compared to MBrCs are shown in red, and miRNAs downregulated in melanoma are shown in blue. Both are indicated by black arrows. Mentions in the literature of functional relevance of the respective miRNA in melanoma or other cancers are depicted by +.

In the following, we analyzed whether these 89 miRNAs have a function in melanoma development. First, we visualized the differential expression analysis of those 89 miRNAs in a heatmap (Figure 2). A major part (63%) of those 89 miRNAs was upregulated in melanoma compared to MBrCs and 33% of the miRNAs were downregulated. In the following chapters, we separately focus on the upregulated and downregulated miRNAs, to elaborate whether they represent a group of regulators that play an important role during tumor progression.

### 3.2. miRNAs Significantly Upregulated in Melanoma Cells Compared to MBrCs and NHEMs Regulate Important Target Genes Driving Tumorigenesis

For some of the miRNAs, which we found most strongly upregulated in melanoma cells compared to MBrCs and NHEMs, a functional role in melanoma development has already been described (see Figure 2, e.g., [42,43]). In addition, most of these miRNAs have known target genes with described functions in other cancers but no melanoma-associated role is known today, for example, miR-105 and miR-4284 [44,45]. Interestingly, miRNAs without known function to date are strongly upregulated in melanoma cell lines.

**Figure 3:**
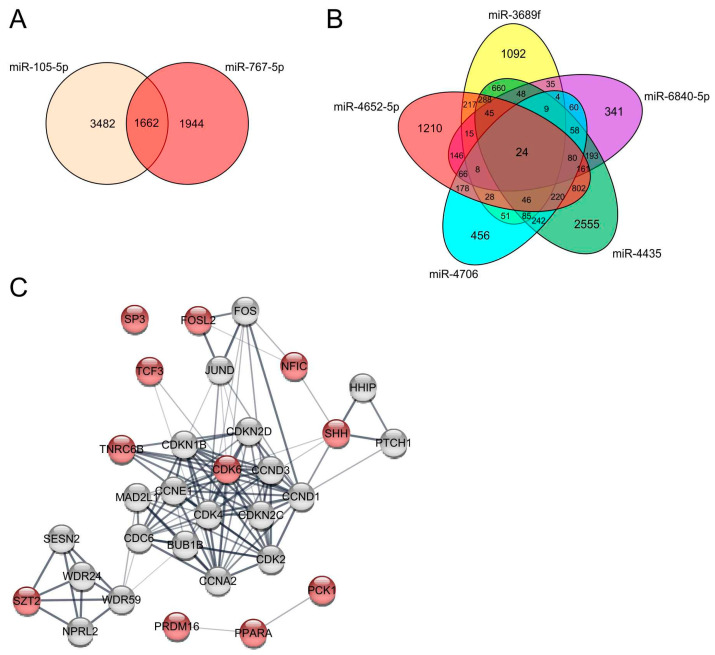
Target gene analysis of strongly upregulated miRNAs in melanoma compared to MBrCs and NHEMs. (**A**) Venn diagram of predicted targets of miR-105-5p and miR-767-5p. (**B**) Venn diagram of predicted target genes of miR-3689f, miR-6840-5p, miR-4652-5p, miR-4706, and miR-4435. (**C**) STRING network analysis of selected target genes of the 24 shared target genes of miR-3689f, miR-6840-5p, miR-4652-5p, miR-4706, and miR-4435 (red), and additional genes involved in the network (grey).

To elucidate the role of the upregulated miRNAs for the development of melanoma more deeply, we examined predicted target genes of the two most strongly upregulated miRNAs in melanoma cells, miR-767-5p and miR-105-5p using targetscan (http://www.targetscan.org/vert_72, release 7.2 March 2018 [31]). miR-767-5p and miR-105-5p share 1662 predicted target genes (Figure 3A). A gene ontology (GO) enrichment analysis of those targets showed, amongst others, a significant enrichment of genes involved in the Wnt signaling pathway (Supplementary Table S1) (http://geneontology.org, release 1 January 2021 [32]).

Other miRNAs that were strongly upregulated in melanoma cells compared to MBrCs and NHEMs, which are not described to have a functional role in melanoma or other cancers yet, were miR-3689f, miR-6840-5p, miR-4652-5p, miR-4706, and miR-4435 (see Figure 2).

A target gene analysis of those five miRNAs provided an enrichment of genes, e.g., involved in neuron development, response to cAMP, homophilic cell adhesion via plasma membrane adhesion molecules, protein localization to the plasma membrane and the cell periphery, actin cytoskeleton organization, regulation of BMP signaling pathway, endothelial cell differentiation, and negative regulation of developmental growth (Supplementary Table S1).

Further, we examined the shared predicted target genes of those five unknown miRNAs (Figure 3B). miR-3689f, miR-6840-5p, miR-4652-5p, miR-4706, and miR-4435 share 24 target genes. We analyzed those targets in a STRING network analysis (https://string-db.org, version 11.0b October 2020 [33]) (Figure 3C). Interestingly, all five miRNAs regulate targets that belong to a network with cyclin-D1 (CCND1), cyclin-E1 (CCNE1), cyclin-dependent kinase 4 inhibitor C (CDKN2C), and cyclin-A2 (CCNA2), and are strongly implicated in G1/S cell cycle transition and cell cycle regulation. Further, many shared target genes of these five miRNAs are important transcription factors such as FOSL2, NFIC, TCF3, and SP3. Additionally, PPARA, SHH, SZT2 were also determined to be shared target genes. PPARA, the peroxisome proliferator-activated receptor alpha, is a central regulator of lipid metabolism and can also function as a transcription factor. SHH, the sonic hedgehog protein, strongly interacts with its receptor PTCH1 and the hedgehog-interacting protein HHIP. The interaction between SHH and PTHC1 plays an important role during tumor growth and the regulation of apoptosis [46]. SZT2 is part of the KICSTOR complex, which regulates the amino acid-sensing part of the mTORC1 signaling pathway being required for many processes during cell proliferation and survival and often deregulated in cancer [47]. In conclusion, shared target genes of miR-3689f, miR-6840-5p, miR-4652-5p, miR-4706, and miR-4435 and their role for cell proliferation and survival implicates a so far unknown role of those five strongly upregulated miRNAs in melanoma development.

To further analyze our second hypothesis that especially genes, which are only deregulated in melanoma development but not differentially expressed during embryonal differentiation drive tumor development, we examined gene expression array data of genes differentially regulated comparing melanoma cells to MBrCs and NHEMs. To detect important miRNAs regulating these genes, gene set enrichment analysis (GSEA) was performed. We used gene sets containing predicted binding sites of miRNAs and compared them to a ranked list of genes from the gene expression array ranging from significantly upregulated genes in melanoma cells compared to MBrCs and NHEMs to significantly downregulated genes.

**Figure 4:**
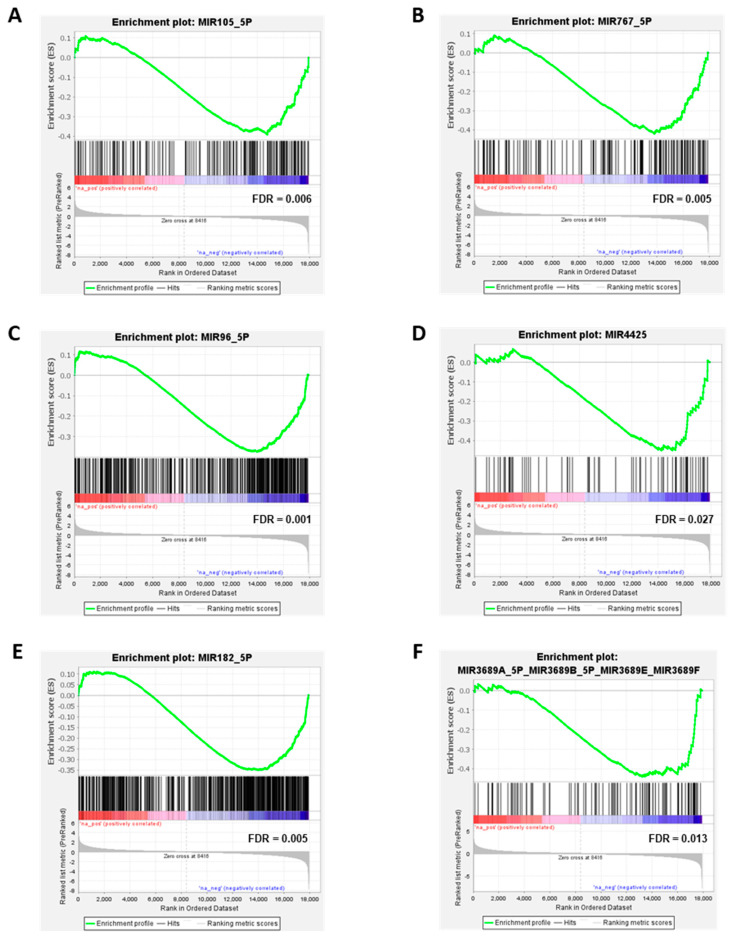
Significant downregulation of miRNA target genes in melanoma cells. Enrichment plot for (**A**) miR-105-5p, (**B**) miR-767-5p, (**C**) miR-96-5p, (**D**) miR-4425, (**E**) miR-182-5p, and (**F**) miR-3689F. Profile of the running enrichment score (green) and positions of gene set members of the respective miRNA target genes on the rank-ordered list of genes differentially regulated in melanoma cells compared to MBrCs and NHEMs identified by gene expression array analysis. Genes upregulated in melanoma are shown on the left side of the graph in red, genes downregulated in melanoma on the right side in blue.

The GSEA analysis reveals that many target genes of the observed upregulated miRNAs are significantly downregulated in melanoma cells compared to MBrCs and NHEMs. The miRNAs miR-767-5p, miR-105-5p, mir-96-5p, miR-4425, miR-182-5p, and miR-3689f were analyzed in more detail (Figure 4).

miR-767-5p and miR-105-5p are the two most strongly upregulated miRNAs in melanoma cells compared to MBrCs (see Figure 2). The GSEA analysis showed a strong downregulation of their target genes in melanoma cells compared to MBrCs and NHEMs (Figure 4A,B) supporting our hypothesis that those miRNAs are important regulators of melanoma development. miR-96-5p, miR-4425, and miR-182-5p are known to regulate essential target genes in other tumor types [48,49,50]. Our data show significant downregulation of target genes of those miRNAs in melanoma cell lines compared to NHEM and MBrC (Figure 4A,C–E) implicating their important role also in melanoma. The role of miR-3689f is so far completely unknown in melanoma or other cancers. We found, on the one hand, a significant and strong upregulation of this miRNA in melanoma cells compared to MBrCs and NHEMs (Figure 2), and on the other hand, a significant downregulation of its target genes in melanoma (Figure 4F).

The similar expression of those miRNAs in NHEMs and MBrCs but strong upregulation in melanoma and the identified significant downregulation of their target genes in melanoma cells strongly supports our hypothesis. Therefore, those miRNAs have a significant share in driving the tumorigenic phenotype of melanoma.

### 3.3. miRNAs Significantly Downregulated in Melanoma Cells Compared to MBrCs and NHEMs Are Implicated in the Regulation of Tumorigenic Pathways

**Figure 5:**
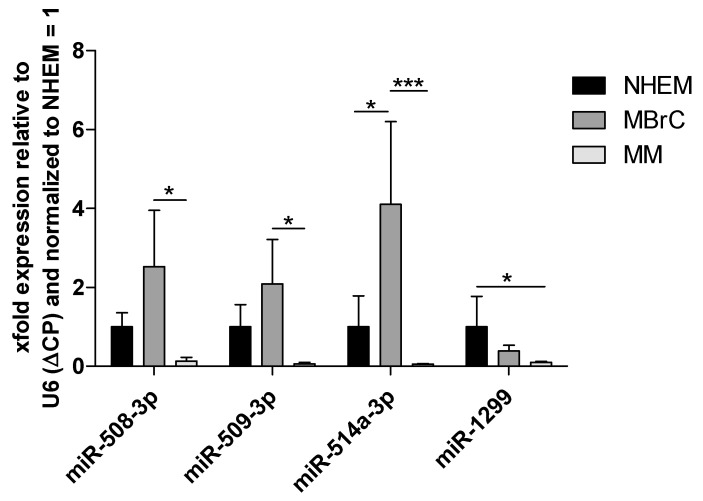
QRT-PCR shows a strong downregulation of miRNAs of the miR-506–514 cluster and miR-1299. Analysis of miR-508-3p, miR-509-3p, miR-514a-3p, and miR-1299 expression in normal human epidermal melanocytes (NHEM), melanoblast-related cells (MBrC), and at least 6 different melanoma cell lines (MM) via qRT-PCR. Bars represent mean + SEM, statistical significance was calculated using one-way ANOVA and subsequent Tukey’s Multiple Comparison Test with ΔCP values before normalization to NHEM and is indicated as * *p* ≤ 0.05 and *** *p* ≤ 0.001.

Focusing on those miRNAs that are significantly downregulated in melanoma vs. MBrCs and NHEMs yields in miRNAs that have already been described as implicated in melanoma development or have described functional roles in other cancers (see Figure 2, e.g., [51,52,53,54]). Interestingly, our analysis discovered many miRNAs of the so-called miR-506–514 cluster (miR-506, miR-508, miR-509, miR-513, and miR-514) as strongly and significantly downregulated in melanoma cells compared to MBrCs and NHEMs. The miR-506–514 cluster has been previously shown to be overexpressed in melanoma patient biopsies and cell lines and to promote melanoma growth [55]. To, therefore, confirm our miRNA expression data obtained by RNA-seq, we additionally performed qRT-PCR-based expression analysis of selected miRNAs of this miRNA cluster (Figure 5).

Significant downregulation of miR-508-3p, miR-509-3p, and miR-514a-3p in melanoma cell lines compared to MBrCs can be observed, confirming the RNA-seq expression results. In addition, we could also show a strong downregulation of miR-1299. These data support the reliability of our RNA-seq analysis.

**Figure 6:**
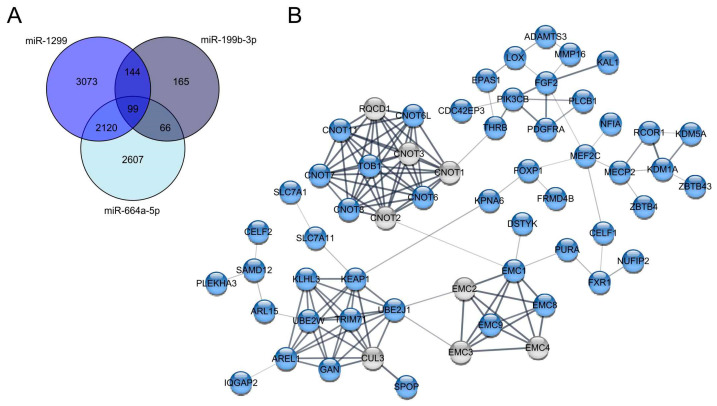
Target gene analysis of strongly downregulated miRNAs in melanoma compared to MBrCs and NHEMs. (**A**) Venn diagram of shared predicted targets of miR-1299, miR-199b-3p and miR-664a-5p. (**B**) STRING network analysis of selected target genes of the 99 shared targets of miR-1299, miR-199b-3p, and miR-664a-5p (blue), which belong to one network and additional genes involved in the network (grey).

Although the most strongly downregulated miRNAs in melanoma compared to MBrCs obtained in our analysis were already described in the literature, most of those miRNAs have only known functional roles in other tumors but no association with melanoma development until now [54,56]. miRNAs with unknown function in melanoma are miR-1299, miR-199b-3p, and miR-664a-5p. We analyzed predicted target genes of those three miRNAs and found 99 shared targets (Figure 6A). GO term analysis of those 99 shared target genes showed significant enrichment of genes involved in stem cell proliferation, embryonic skeletal system morphogenesis, embryonic skeletal system development, and negative regulation of translation (Supplementary Table S1).

Additional 144 shared targets of miR-1299 and miR-199b-3p include genes involved in developmental growth and embryo development.

Additionally, we analyzed the 99 shared targets of miR-1299, miR-199b-3p, and miR-664a-5p in a STRING network analysis to get an overview of how the target genes are related to each other (Figure 6B). The STRING analysis of the targets shows several clusters of connected genes.

The first gene cluster includes general CCR4-NOT transcription complex subunits (CNOT), which are involved in gene silencing by miRNA, transcriptional regulation, and negative regulation of the cell cycle [57]. The next target gene cluster includes the endoplasmic reticulum (ER) membrane protein complex subunits (EMC1-4, 8, and 9). These genes are known to be involved in protein folding. Another target gene cluster consists of the following genes: KLHL3, KEAP1, UBE2W, UBE2J1, TRIM71, AREL1, SPOP, CUL3, and GAN (Figure 6B). CUL3, KEAP1, and TRIM 71, for example, are involved in embryonic development. Additionally, some of these proteins are involved in proteasomal degradation via ubiquitination. The next cluster contains the genes KDM5A, KDM1A, ZBTB43, ZBTB4, MECP2, and RCOR1. These genes have the molecular function of transcription regulator activity in common. Moreover, KDM1A, KDM5A, and MECP2 are involved in embryonic development. The genes in the last cluster, PLCB1, PIK3B, PDGFRA, FGF2, and KAL1, are involved in developmental processes as well, with FGF2, PDGFRA, and PIK3CB being involved in embryonal development. Moreover, FGF2, PDGFRA, and PIK3CB play a role in the regulation of the actin cytoskeleton. Interestingly, FGF2 and PIK3CB are known to be involved in the activation of the mitogen-activated protein kinase (MAPK) activity. In summary, the STRING analysis provides a reliable overview of potentially important target genes that miR-1299, miR-199b-3p, and miR-664a have in common. The loss of these three miRNAs and the resulting upregulation of the described target genes might play a significant role in melanoma development and progression.

### 3.4. miRNAs Differentially Expressed in Only Metastasis-Derived Melanoma Cell Lines Compared to Melanoblasts Provide Information about Metastasis Processes

**Figure 7:**
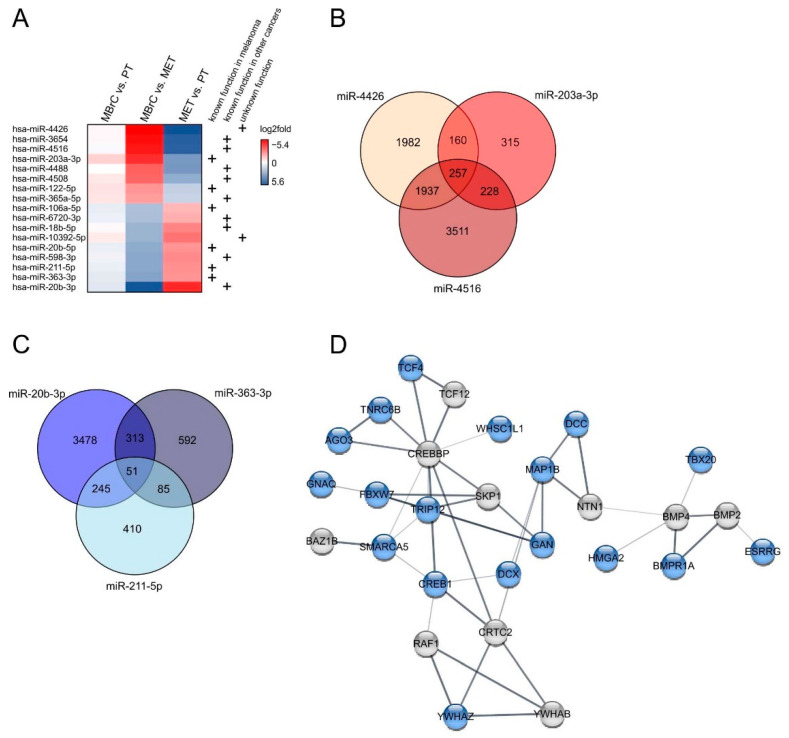
miRNAs are significantly regulated in only metastasis-derived melanoma cell lines. (**A**) miRNAs that are not regulated in MBrCs vs. NHEMs (*p* > 0.05) and only significantly differentially expressed (*p* < 0.05) in MBrCs vs. melanoma metastasis-derived (MET) cell lines with log2fold in MET vs. PT > 1 or < −1 and in MBrC vs. PT > −1 and simultaneously < 1. Mentions in the literature of functional relevance of the respective miRNA in melanoma or other cancers are indicated (+). (**B**) Venn diagram of shared predicted targets of upregulated miR-4426, miR-203a-3p, and miR-4516. (**C**) Venn diagram of shared predicted targets of downregulated miR-20b-3p, miR-363-3p, and miR-211-5p. (**D**) STRING network analysis of connected target genes of the 51 shared targets of miR-20b-3p, miR-363-3p, and miR-211-5p (blue) and additionally involved genes (grey).

As previously shown, there are 89 miRNAs, which are significantly regulated during early tumor development (primary tumor cell lines) and stay highly expressed during tumor progression (metastasis-derived cell lines). The analysis also resulted in 61 miRNAs that are significantly differentially expressed (*p*-value < 0.05) only in MBrCs vs. metastasis-derived melanoma cell lines (see Figure 1B,C). Further focusing on these 61 miRNAs, based on strong miRNA regulation only in metastasis-derived cell lines (log2fold MET vs. PT > 1 or < −1) and absence of regulation during early tumorigenesis (log2fold MBrC vs. PT > −1 and simultaneously < 1), resulted in 17 miRNAs fulfilling the criteria (Figure 7A). Several of those miRNAs are already known to play a role during the development and metastasis of melanoma or other cancers (see Figure 7A, e.g., [58,59,60]). To determine if unknown miRNAs of this list (Figure 7A) have a specific impact on tumor metastasis, we analyzed those miRNAs in more detail.

Predicted target genes of the most strongly upregulated miRNA, miR-4426, which is so far unknown in cancer (Figure 7A), are implicated in the canonical Wnt signaling pathway, positive regulation of locomotion, positive regulation of cell migration, and positive regulation of cell motility (Supplementary Table S1).

miR-4426 shares 417 targets with miR-203a-3p (Figure 7B), a miRNA known in melanoma as a biomarker for metastatic melanoma [61], but the functional role of this miRNA is unknown so far. These target genes are involved in the regulation of transmembrane receptor protein serine/threonine kinase signaling pathway, cell population proliferation, and positive regulation of cell differentiation (Supplementary Table S1). Targets of miR-203a-3p alone are related to the BMP signaling pathway, mesenchymal cell development, and neural crest cell differentiation.

Further, miR-4426 shares 2194 predicted target genes with miR-4516 that are supposed to be important for positive regulation of blood vessel endothelial cell migration.

miR-4426, miR-4516, and miR-203-3p share 257 targets, and some of them are significantly involved in the regulation of developmental processes, internal protein amino acid acetylation, and histone acetylation implicating a role of these miRNAs into epigenetic regulatory processes.

The most strongly downregulated miRNA in metastasis-derived cell lines compared to primary tumor cell lines and MBrCs is miR-20b-3p (Figure 7A). Predicted target genes of this miRNA are implicated in cell cycle and nervous system development (Supplementary Table S1). miR-363-3p, which is also strongly downregulated, has predicted target genes that are involved in actin polymerization-dependent cell motility and adherens junction assembly.

miR-20b-3p and miR-363-3p share 51 predicted target genes with miR-211-5p (Figure 7C) that are significantly involved in neuron migration. The strong downregulation of those three miRNAs in only metastasis-derived melanoma cell lines indicates a specific role of those miRNAs in migratory processes. A STRING network analysis of selected target genes of those 51 shared targets shows 28 interacting genes (Figure 7D). The genes CREB1, RAF1, YWHAB, YWHAZ, and CRTC2 are involved in the KEGG pathway of PI3K-Akt signaling. It is known that dysregulation of the PI3K-Akt signaling pathway is related to a poor treatment outcome in melanoma [62]. Additionally, CREBBP and SKP1 are involved in the TGF-beta signaling pathway. TGF-beta is known to play a role in melanoma progression and growth [63]. Moreover, target genes of miR-20b-3p, miR-363-3p, and miR-211-5p strongly interact with BMP4 and BMP2, which are involved in the positive regulation of the epithelial to mesenchymal transition. In summary, the string analysis implicates that target genes of miR-20b-3p, miR-363-3p, and miR-211-5p are involved in important processes of melanoma metastasis and progression.

## 4. Discussion

In this study, we examined the expression profile of miRNAs in various stages of development from melanoblasts to melanocytes to melanoma. RNA-sequencing and subsequent bioinformatical analyses revealed that miRNAs are mainly equally expressed in embryonic development from MBrCs to NHEMs, but strongly deregulated during the development of melanoma. We hypothesize that those miRNAs are the key drivers of malignancy as they account for the tumorigenic potential that differentiates melanoma cells from proliferating or migrating embryonic cells.

Several of those miRNAs identified in our study are already well described in the literature as important regulators of melanoma development. An example is some members of the well-characterized let-7 miRNA-family, which we found as strongly downregulated in melanoma cells compared to MBrCs and NHEMs. It is known that let-7 miRNAs function as important tumor suppressors in melanoma [64]. An overexpression in melanoma of, e.g., let-7b leads to a decreased level of cell cycle regulators like cyclin D1 and cyclin D3 [65]. Moreover, the loss of let-7a leads to an upregulation of ITGB3 indicating involvement in melanoma development and progression [66]. Further, miR-1246, which we found as strongly upregulated in melanoma cells in our study, is known to act as an oncogene in melanoma via influencing cell viability and migration and promoting BRAF inhibitor resistance [43,67]. The differential expression analysis in our study confirmed the regulation of those prominent tumor-promoting miRNAs in melanoma indicating the reliability of our gained results.

In addition, our study also showed a strong downregulation of the miRNAs miR-506, miR-508, miR-509, miR-513, and miR-514, which belong to a miRNA cluster called miR-506–514. This cluster has been previously shown to be overexpressed in melanoma patient biopsies and cell lines and to promote melanoma growth [55]. miR-514a-3p was further described to regulate the tumor suppressor NF1 and to be involved in BRAF inhibitor sensitivity in melanoma [68]. Interestingly, in our study, many miRNAs belonging to the miR-506–514 cluster are strongly downregulated in melanoma cells compared to MBrCs and NHEMs. The downregulation could be confirmed via qPCR in independent samples, implicating an interesting new and controversial role of this miRNA cluster in melanoma development.

Many of the miRNAs, which we detected as significantly regulated in melanoma, are already described in the literature as relevant for the development of other cancers [69,70] or are known to be dysregulated in melanoma, but without a distinctly described molecular function [17,71]. The analysis of their potential target genes and involved pathways in our study allows interesting conclusions to be drawn about their role in melanoma tumorigenesis. For example, miR-767 and miR-105, the two most strongly upregulated miRNAs in melanoma cells in our study, both belong to a group of genes, normally inactivated by DNA methylation in somatic tissues [72]. Hypomethylation during cancer development can lead to activation of such genes whose expression is normally restricted to embryonic development, so-called “cancer-germline” genes. miR-767 and miR-105 have been shown to be genomically located in a “cancer germline” gene that is aberrantly activated in melanoma cell lines and tissues [72]. Additionally, miR-767-5p is also upregulated in different other cancer types such as thyroid cancer or hepatocellular carcinoma [73,74]. Further, our study shows a strong downregulation of target genes of miR-767 and miR-105 in melanoma cells. It is known from the literature that miR-767 drives tumorigenesis in other cancers via repression of the tumor suppressors TET1 and TET3, which are responsible for the transformation of 5-methylcytosines to 5-hydroxymethylcytosines (5hmC) in DNA [72]. It is known from pancreatic cancer that a low TET1 expression and subsequent low 5hmC content lead to low survival of patients because of high tumor cell proliferation and metastasis [75]. This is due to loss of the TET1 mediated inhibition of both the canonical and non-canonical Wnt signaling, which leads to increased EMT in tumors with low TET1 expression [75]. A possible misregulation of the Wnt signaling pathway was also observed in this study for shared putative target genes of miR-767 and miR-105. For melanoma, it is also known that the Wnt signaling pathway is often dysregulated [76]. Additionally, Wnt signaling plays a role in the migration of neural crest cells (NCC) and multipotent precursor cells [77,78]. In our study, regulation of target genes associated with Wnt signaling was also observed for several other miRNAs, which are significantly regulated in only metastasis-derived melanoma cell lines, such as miR-4426, miR-203a-3p, and miR-211-5p. This indicates that those genes play an important role throughout the whole process of tumor development and are selectively regulated by numerous miRNAs in different tumor stages.

The results indicate that the newly detected deregulated miRNAs in our study play a significant role during both melanoma development and metastasis. The connection of those miRNAs with the Wnt signaling pathway is so far not described and can be a promising target for future analyses.

Next to the Wnt signaling pathway, our study discloses many other important signaling pathways where targets of the analyzed deregulated miRNAs are involved in. For example, in melanoma cells strongly upregulated miR-3689f, miR-6840-5p, miR-4652-5p, miR-4706, and miR-4435 share the target FOSL2, a transcription factor that dimerizes with JUN and binds to AP-1 sites. It is known from other cancers, that FOSL2 is activated by the ERK1/2 kinase leading to the transcription of SNAI2, which plays an important role in tumor metastasis via activation of epithelial-to-mesenchymal transition (EMT) [79]. Further target genes involved in EMT have been observed for many other miRNAs shown as dysregulated in melanoma cells in our study, underlining the important role of this process in tumor development and especially for tumor cell migration.

In addition, our study shows a regulation of miR-20b-3p, miR-363-3p, and miR-211-5p in only metastasis-derived melanoma cells (see Figure 7C,D) leading to an upregulation of, amongst others, BMP4 and BMP2. BMP4 is known to be regulated by LIF (leukemia inhibitory factor), which might be involved in melanoma-induced bone metastasis [80,81]. It is also known from the literature, that in melanoma the establishment of drug resistance has some signaling pathway activation in common with cell lineage development, e.g., genes associated with the ERK1/2 or the BMP signaling pathway [82]. In our study, many identified regulated miRNAs are related to these pathways, therefore, it can be concluded that the MBrC in vitro model is not only useful to detect alterations in miRNA expression according to melanoma development but may also improve comprehending the expression profiles of drug-resistant melanomas.

For some of the newly identified miRNAs in our study, which are deregulated only in melanoma cells compared to MBrCs and NHEMs, a distinct connection of their associated target genes with specific developmental processes was observed. For example, for the strongly downregulated miR-1299, miR-199b-3p, and miR-664a-5p, which have no described function in melanoma so far. The results indicate that a loss of those miRNAs during melanoma development could result in a dysregulation of fine-tuning of developmental genes that regulate normal embryonic development and may lead to uncontrolled proliferation.

Thus, our study revealed many significantly regulated miRNAs, which are related to important pathways involved in melanoma development and progression. The connection between the newly identified miRNAs and their target genes could be the main contributor to the tumorigenicity of melanoma, which has not been investigated so far.

Many properties of embryonic cells, such as proliferation, migration, and invasion also play an important role during tumorigenesis and especially in metastasis. In our study, we examined miRNAs, which are only regulated in metastasis-derived melanoma cell lines compared to MBrCs and NHEMs to detect target genes and mechanisms that may drive the metastatic phenotype. We identified in total 89 miRNAs being significantly regulated in primary tumor and metastasis-derived cell lines. In contrast, only a few miRNAs were significantly regulated in metastasis-derived cell lines but not in primary tumor cell lines. This indicates that the most important changes leading to melanoma tumorigenesis on the miRNA level seem to already take place at the beginning of tumor development. Interestingly, our study revealed that many processes being regulated by targets of miRNAs that are upregulated in metastasis-derived melanoma cells, such as the Wnt signaling pathway or the BMP signaling pathway. These are also regulated by other miRNAs, which are already upregulated in primary tumor-derived melanoma cells. This indicates that those pathways play an important role throughout the whole process of tumor development and are selectively regulated by numerous miRNAs in different tumor stages.

## 5. Conclusions

This study revealed a strong and significant regulation of many miRNAs in melanoma cell lines compared to healthy cells but a similar expression profile of miRNAs during melanoblast to melanocyte development. Our analyses support our hypothesis that those miRNAs drive tumorigenesis and represent the decisive difference to comparable processes of proliferation or migration during embryonic development. Following this hypothesis, we identified many miRNAs, which were not described during melanoma development so far, but whose target gene analysis and comparison to known target genes from other cancers implicate a promising role as tumor driving candidates. This study lays the foundation for further interesting analyses of those novel miRNAs that may play a crucial role in the development and progression of malignant melanoma.

## Data Availability

miRNA-sequencing data are deposited in the Gene Expression Omnibus (GEO) database under the accession number GSE174334.

## Supplementary Materials

The following supplementary materials are available online at https://www.mdpi.com/article/10.3390/jcm10112259/s1, Table S1: Gene ontology enrichment analysis of miRNA target genes.
